# Host serum miR-223 is a potential new biomarker for *Schistosoma japonicum* infection and the response to chemotherapy

**DOI:** 10.1186/1756-3305-6-272

**Published:** 2013-09-20

**Authors:** Xing He, Xue Sai, Chao Chen, Yuanbin Zhang, Xindong Xu, Dongmei Zhang, Weiqing Pan

**Affiliations:** 1 Department of Tropical Infectious Diseases, Second Military Medical University, Shanghai 200433, China; 2 Institute for Infectious Diseases and Vaccine Development, Tongji University School of Medicine, Shanghai 200411, China

**Keywords:** Schistosomiasis, Serum miRNA, Biomarker, *Schistosoma japonicum*, Praziquantel

## Abstract

**Background:**

Numerous studies have shown that aberrant microRNA (miRNA) expression is associated with the pathogenesis and progression of various human diseases. Hence, serum miRNAs are considered to be potential biomarkers for the diagnosis of human diseases. This study examined whether several miRNAs known to be commonly deregulated in liver diseases are deregulated in the serum of hosts with hepatic schistosomiasis, and thus whether they could serve as potential markers for detection of schistosome infection and evaluation of the effectiveness of chemotherapy.

**Methods:**

We analyzed the serum levels of six selected candidate miRNA molecules (miR-146b, miR-122, miR-223, miR-199a-5p, miR-199a-3p, miR-34a) from mice, rabbits, buffalos and humans infected with *Schistosoma japonicum* using qPCR. We evaluated liver pathology by determining the hydroxyproline content in liver tissues. Primary resident liver cells were isolated to quantify the expression level of deregulated miRNAs. Bioinformatics analyses were also conducted to assess the potential function of miR-223.

**Results:**

Using a mouse model of *Schistosoma japonicum* infection, we found that the expression level of serum miR-223 was significantly elevated after infection, but returned to near normal levels after the treatment with praziquantel (PZQ). Importantly, the level of serum miR-223 reflected the extent of liver pathology post-infection. We validated the elevated level of the circulating miR-223 in serum samples of other host species including rabbits, buffalos and humans. In addition, our results showed that miR-223 was primarily located in the Kupffer cells, but its expression levels were significantly up-regulated in hepatocytes, hepatic stellate cells and Kupffer cells after infection. Bioinformatics analyses revealed a potential functional role of miR-223 in transcription regulator activity, transcription factor activity and DNA binding.

**Conclusions:**

This study suggested that the circulating miR-223 could serve as a potential new biomarker for the detection of schistosome infection and the assessment of the response to chemotherapy.

## Background

Schistosomiasis is a serious tropical parasitic disease caused by infection with helminths in the genus, *Schistosoma*, which affects more than 200 million people worldwide [1,2]. Of the three schistosome species that infect humans, only *Schistosoma japonicum* (*S. japonicum*) is endemic in China, where it constitutes a major public health problem [3,4]. The primary cause of mortality from schistosomiasis japonica is liver fibrosis, which results in portal hypertension and variceal bleeding [1]. For more than 30 years, treatment of schistosomiasis japonica has involved administering the anti-schistosome drug praziquantel (PZQ) [5,6]. The high prevalence of the disease is partially due to the lack of effective detection tools for diagnosis and response to chemotherapy. At present, however, there are no reliable biomarkers to enable the diagnosis of schistosomiasis infections, or to facilitate detection of the response to the chemotherapy by PZQ.

MicroRNAs (MiRNAs) are a class of highly conserved, small, non-coding RNA molecules that regulate gene expression post-transcription [7-9]. Numerous studies have shown that aberrant miRNA expression is associated with various types of human diseases including liver diseases [10]. For example, miR-146b and miR-34a were up-regulated in the liver tissues of patients with non-alcoholic steatohepatitis [11], while in the CCl_4_ induced liver fibrosis, miR-199a-5p and miR-199a-3p were positively and significantly correlated to the progression of liver fibrosis [12]. Recently, miRNAs were shown to play important roles in mediating the host-schistosome interaction in a non-permissive host environment [13], and in moderating regulatory T-cell function during schistosome infection [14].

As cell-free miRNAs in serum are very stable under harsh conditions, such as low or high pH, boiling, extended storage, and multiple freeze-thaw cycles, they are considered important new blood-based biomarkers for disease diagnosis and prognosis [15-17]. In addition, the expression profiles of serum miRNAs are altered during pathological processes, providing another valuable trait for their use as a disease biomarker. For example, serum miR-21, miR-122 and miR-223 are elevated in patients with hepatocellular carcinoma and chronic hepatitis and thus, have the potential to serve as novel biomarkers for liver injury [18]. Previous studies have also shown that circulating miRNA-146a and miR-223 were significantly reduced in septic patients [19], while serum miRNA-122 and miRNA-192 were elevated in a mouse model of drug-induced liver injury [20]. The alteration of miRNA expression profiles in serum of mice or patients with schistosomiasis japonica is still largely unknown.

In this study, we hypothesized that some common miRNAs frequently observed to be deregulated in human liver diseases might be deregulated in the serum of hosts with schistosomiasis japonica, and could thus serve as novel biomarkers for the detection of schistosome infection. To test this hypothesis, we selected six candidate serum miRNAs for analysis (miR-146b, miR-122, miR-223, miR-199a-5p, miR-199a-3p, miR-34a) in the murine model of human schistosomiasis and then performed validation in other host species including rabbits, buffalos and human patients infected with *S. japonicum.*

## Methods

### Schistosome infection and sample preparation

Six-week-old male BABL/c mice were purchased from the experimental animal center of the Second Military Medicine University. Mice were exposed percutaneously to 16 *S. japonicum* cercaria that were shed from lab-infected snails (*Oncomelania hupensis*) obtained from the National Institute of Parasitic Disease, Chinese Center for Disease Control and Prevention. At 42 days post-infection, a time when hepatic fibrosis becomes obvious, half of the mice were treated with PZQ for three days (PZQ treated group) and half were not treated (untreated group). Uninfected BALB/c mice of the same age were also used in the experiment (uninfected group). All the mice were sacrificed at 72 days post-infection to harvest blood serum and liver samples.

We also conducted a separate experiment to determine the expression level of serum miR-223 in the progression of mouse schistosomiasis. To this end, mice were infected with 16 *S. japonicum* cercariae, and sacrificed at 0, 42, 56, 70 days post-infection to harvest the serum and liver samples.

*S. japonicum* can infect many other animals including buffalo that are the primary infection source of transmission in China [3]. Therefore, we analyzed the expression level of miR-223 in serum of other hosts of schistosomes, including rabbits, buffalos and humans. Rabbits were infected percutaneously with 200 *S. japonicum* cercariae or not infected as control. All the rabbits were sacrificed at 56 days after infection to quantify the miR-223 expression level in the sera. Serum samples of schstosomiasis patients diagnosed by parasitological detection were obtained from the field, and uninfected serum samples were obtained from non-endemic areas as a control. All procedures performed on animals in this study were conducted in accordance with, and under approval of, the Second Military Medicine University regulations.

### Egg and parasite counting

The number of schistosome eggs in the liver was counted after the liver tissue was digested by 4% KOH. Liver egg burdens were expressed as 10^4^ eggs per gram of liver tissue. Perfusions of the hepatic portal system were performed to detect the number of adult worms as described [21].

### RNA extraction and reverse transcription

Blood samples were collected from hosts and centrifuged at 3,000 × *g* for 10 min at room temperature to completely remove cellular components, and the supernatant (serum) was retained. RNA was then extracted from 100 μL of serum using the miRNeasy mini kit (Qiagen) according to the manufacturer’s protocol. The *C. elegans* spiked-in control miRNA, cel-miR-39, was used to normalize the technical variability of the serum RNA extraction [22]. Reverse transcription (RT) reactions were performed using the First-Strand cDNA Synthesis Kit (TaKaRa, Dalian, China) and miRNA-specific stem-loop primers in a 10 μL RT reaction: 2 μL H_2_O, 2 μL buffer, 1 μL primer, 0.5 μL dNTPs (10 mM), 0.25 μL RRI, 0.25 μL M-MLV and 4 μL RNA. RT reactions were conducted in a Veriti 96-well thermal cycler (ABI) under the following conditions: 16°C for 30 min, 42°C for 30 min, 85°C for 5 min (then hold at 4°C). RT products were stored undiluted at 20°C prior to running the real-time PCR reactions.

### Quantification of serum miRNAs

Quantification of serum miRNAs was performed according to the qPCR protocols as described previously [23]. The expression levels of miR-34a, miR-223, miR-122, miR-146b, miR-199a-5p, miR-199a-3p were determined using the SYBR Green Master Mix kit (TaKaRa, Dalian, China). Cel-miR-39 was used as an internal control, and the fold change was calculated by the 2^-ΔΔCt^ method [24]. The sequences of the primers used are shown in Table 1.

**Table 1:** Primers used in qPCR

**Gene**	**Primer sequence (5'-3')**	
mmu-miR-223	Forward	ATGGTTCGTGGG TGTCAGTTTGTCAAAT
	Reverse	GCAGGGTCCGAGGTATTC
mmu-miR-146b	Forward	ATGGTTCGTGGGTGAGAACTGAATTCCA
	Reverse	GCAGGGTCCGAGGTATTC
mmu-miR-199a-5p	Forward	ATGGTTCGTGGGCCCAGTGTTCAGACTAC
	Reverse	GCAGGGTCCGAGGTATTC
mmu-miR-199a-3p	Forward	ATGGTTCGTGGGACAGTAGTCTGCACAT
	Reverse	GCAGGGTCCGAGGTATTC
mmu-miR-122	Forward	ATGGTTCGTGGGTGGAGTGTGACAATGG
	Reverse	GCAGGGTCCGAGGTATTC
mmu-miR-34a	Forward	ATGGTTCGTGGGTGGCAGTGTCTTAGCT
	Reverse	GCAGGGTCCGAGGTATTC
cel-miR-39	Forward	ATGGTTCGTGGGTCACCGGGTGTAAATC
	Reverse	GCAGGGTCCGAGGTATTC
U6	Forward	GCTTCGGCAGCACATATACTAAAAT
	Reverse	CGCTTCACGAATTTGCGTGTCAT

### Hydroxyproline content assay

Hepatic fibrosis is the most important pathological change of schistosomiasis, and the elevated hydroxyproline content in the liver is an informative biomarker for hepatic fibrosis. Therefore, we assessed hepatic hydroxyproline content to examine liver pathology. Hydroxyproline content was detected using a colorimetric assay according to the manufacturer’s instructions (Nanjing Jiancheng Bioengineering Institute, Nanjing, China).

### Isolation of mouse hepatic stellate cells (HSCs), hepatocytes and Kuppfer cells

Liver samples were initially digested *in situ* with 0.04% collagenase type IV. They were then further digested with 0.08% collagenase type IV while shaken for 30 minutes in a 37°C bath. The resulting cell suspension was centrifuged at 50 × *g* for 4 minutes to isolate hepatocytes. HSCs were isolated according to the method as described previously [25]. The purity of HSCs was higher than 95% as determined by retinoid autofluorescence. Kuppfer cells were isolated using positive selection with magnetic CD11b antibody beads and magnetic columns (MACS, Miltenyi, Auburn, CA) [26]. FACS-Calibur analysis demonstrated >90% purity after labeling purified cells with CD11b-FITC antibody (Miltenyi; Auburn, CA).

### Gene ontology (GO) analysis

GO analysis was used to analyze the potential function of miRNAs [27]. A two-tailed Fisher’s exact test and a *χ*^2^ test were used to classify the GO category. The false discovery rate (FDR) was calculated to correct the *P*-value. We chose only GOs that had a *P*-value of <0.001 and a FDR of <0.05.

### Statistical analyses

All results are reported as means ± standard deviations and compared between groups using two-tailed Student’s *t*-test or one-way ANOVA. Data were considered statistically significant at *P* < 0.05.

## Results

### Changes of hydroxyproline content in liver tissues

**Figure 1:**
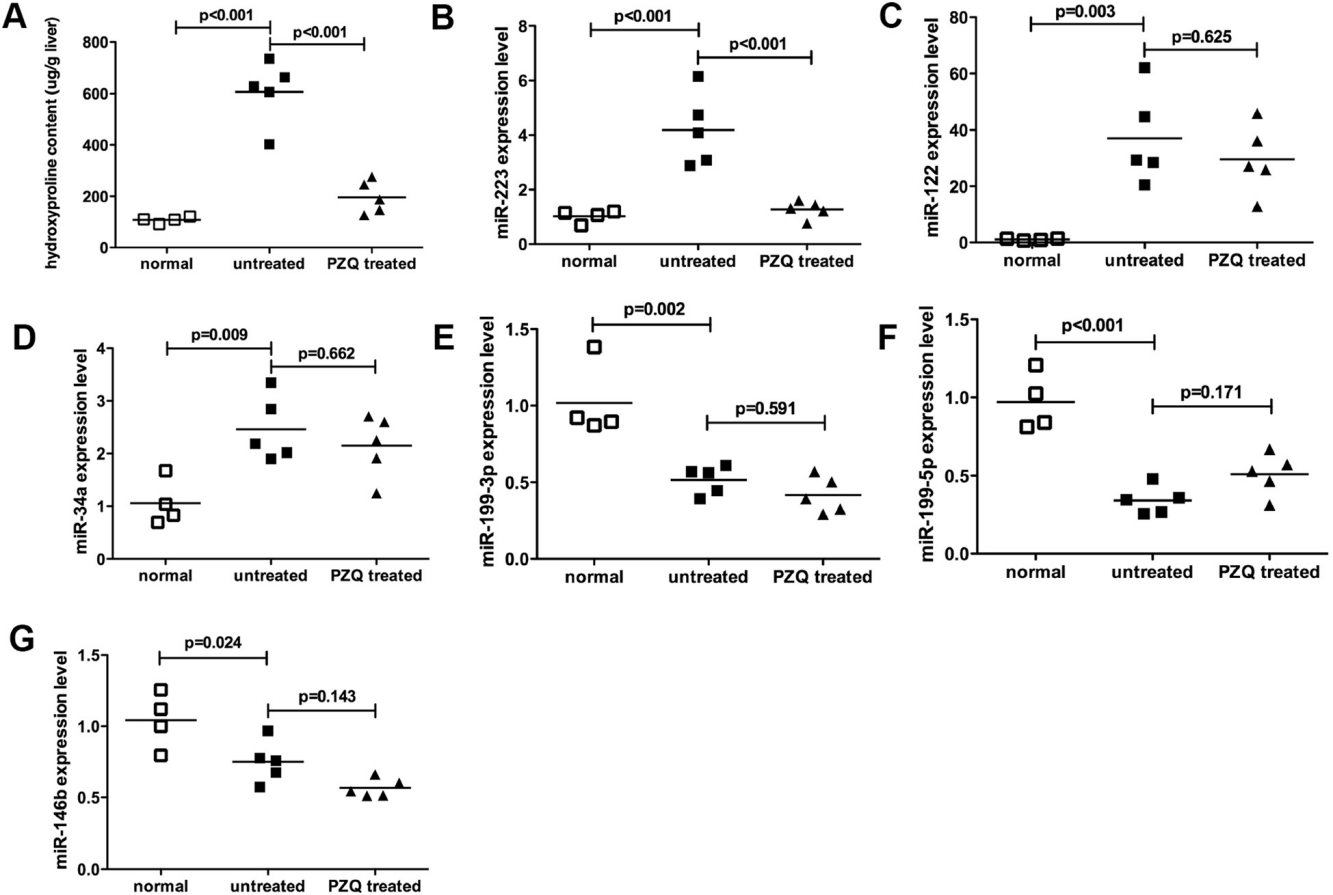
**Alteration of miRNA expression profiles in serum of infected mice with schistosomiasis japonica.** Mice were infected percutaneously with 16 *S. japonicum* cercariae or remained uninfected (normal group, blank square). Half of the infected mice were treated with PZQ for 3 days (PZQ treated group, black triangle) and half were not treated (untreated group, black square). All the mice were sacrificed at 72 days after infection to harvest the serum and liver samples. **(A)** Hydroxyproline content of liver samples of mice from the three groups was detected. Expression levels of serum miR-223 **(B)**, miR-122 **(C)**, miR-34a **(D)**, miR-199a-5p **(E)** miR-199a-3p **(F)**, and miR-146b **(G)** were detected in the three groups of mice. The data shown are measurements from individual mice. The bars designated the means for each group.

**Figure 2:**
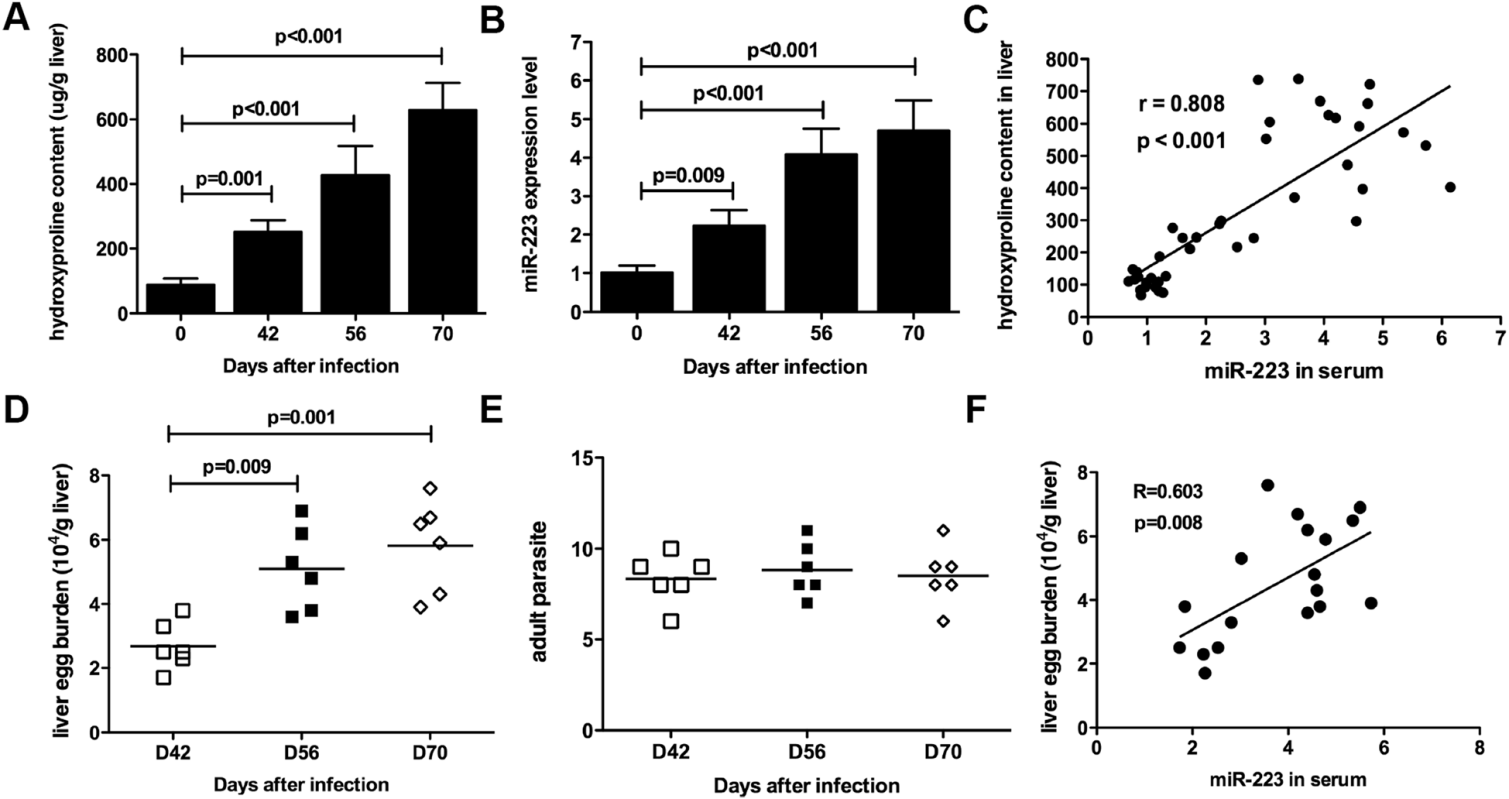
**Correlation between serum miR-223 expression level and liver hydroxyproline content in the infected mice.** Mice were infected percutaneously with 16 *S. japonicum* cercariae, and sacrificed at 0, 42, 56, 70 days after infection to harvest the serum and liver samples (n = 6). Hydroxyproline content of liver samples **(A)** and miR-223 expression level of serum samples **(B)** from the four groups were determined, respectively. **(C)** Correlation analysis between the liver hydroxyproline content the serum miR-223 expression level was performed. Eggs trapped in the liver tissue **(D)** and adult parasites living in the mesenteric veins **(E)** were counted. **(F)** Correlation analysis between the liver egg burden and the serum miR-223 expression level was performed.

Hepatic fibrosis is the most important pathological change of schistosomiasis while the elevated hydroxyproline content in the liver is an informative biomarker for hepatic fibrosis. Therefore, we assessed hepatic hydroxyproline content to examine liver pathology. As shown in Figure 1A, hydroxyproline content in the liver tissue of mice in the untreated group increased significantly after infection. After treatment with PZQ, however, the liver pathology was significantly alleviated and returned to near normal level (see results for the treated group; Figure 1A). In addition, we measured the hydroxyproline content at various time points post infection and showed that the hydroxyproline content in the liver of infected mice was increased with the time extended (Figure 2A).

### Expression levels of miRNAs in sera of mice with schistosomiasis

To identify miRNAs that reflected the schistosome infections and PZQ chemotherapy, six miRNA candidates (miR-146b, miR-122, miR-223, miR-199a-5p, miR-199a-3p, miR-34a) were selected for analysis in serum that were commonly deregulated in human liver diseases. In mouse hosts, quantitative PCR result revealed that circulating miR-223, miR-122 and miR-34a were significantly elevated after infection (Figure 1B-D). Conversely, levels of serum miR-199a-3p, miR-199a-5p, and miR-146b in mice decreased after infection (Figure 1E-G). Only one serum miRNA in infected mice, however, decreased significantly after the PZQ treatment (miR-223, Figure 1B).

### Expression level of circulating miR-223 in the progression of schistosomiasis

Next, we analyzed the expression level of serum miR-223 in the progression of mouse schistosomiasis. To this end, mice were infected with 16 *S. japonicum* cercariae, and sacrificed at 0, 42, 56 and 70 days after infection. As expected, the level of circulating miR-223 increased significantly during the course of infection (Figure 2B). Importantly, the level of serum miR-223 was significantly correlated with the hydroxyproline content in the liver tissue (r = 0.808, *P* < 0.001), which suggests that the level of serum miR-223 could reflect the extent of liver pathology after infection (Figure 2C). To investigate any correlation of the serum miR-223 level with the infection intensity, we counted the liver eggs and the adult worms at various time points post infection. As shown in Figure 2D and 2E, the liver egg burden was gradually increased with the time extended, but the adult parasites living in the mesenteric veins remained constantly at the three time points. Moreover, the serum miR-223 level was significantly correlated with the liver egg burden (r = 0.603, *P* = 0.008, Figure 2F).

### Expression level of circulating miR-223 in human and other animal models with schistosomiasis

**Figure 3:**
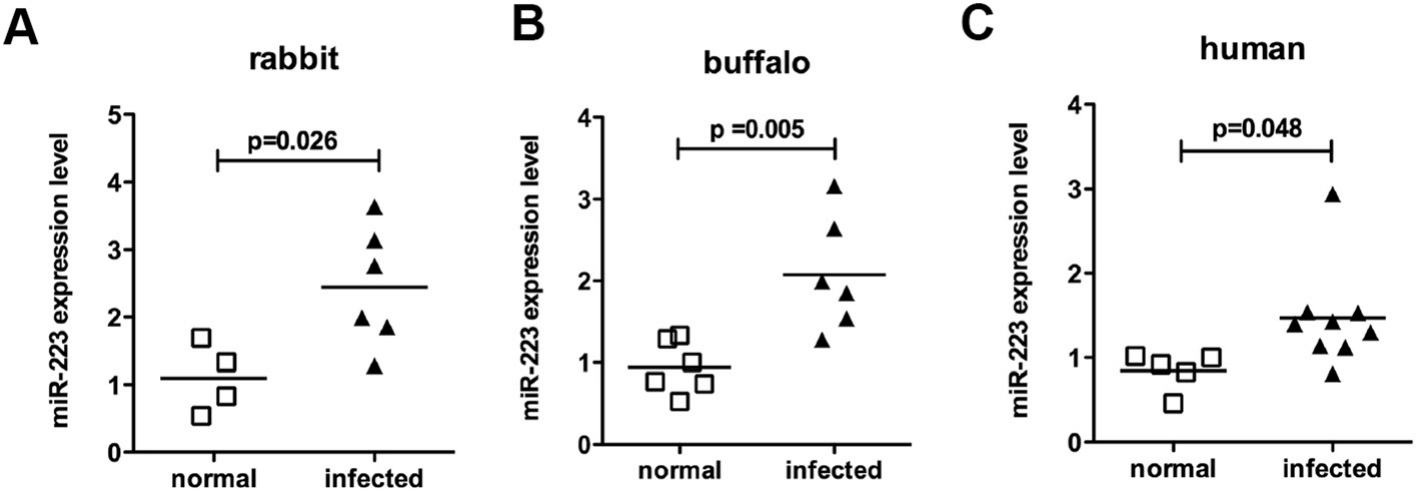
**Expression level of the serum miR-223 in rabbit, buffalo and human with schistosomiasis. (A)** Rabbits were infected percutaneously with 200 *S. japonicum* cercariae or not infected as control. All the rabbits were sacrificed at 56 days after infection to quantify the miR-223 expression level in the sera. **(B**, **C)** The expression level of serum miR-223 was detected in serum samples of schistosomiasis patients or buffalos diagnosed by parasitological detection and uninfected individuals as control. The data shown are measurements from individuals. The bars designate the means for each group.

Schistosomiasis japonica is a zoonotic parasitic disease. In addition to mice, *S. japonicum* can infect other animals such as rabbit, buffalo etc. The latter is the primary infection source of *S. japonicum* transmission in China [3]. Therefore, we analyzed the expression level of miR-223 in serum of other host species of schistosome, including rabbits, buffalos and humans. As shown in Figure 3, the miR-223 expression level was obviously elevated in the serum of infected rabbit, buffalo and human, which validated the results of the miR-223 obtained from the mouse model of human schistosomiasis.

### Expression level of miR-223 in resident liver cells and its potential function

**Figure 4:**
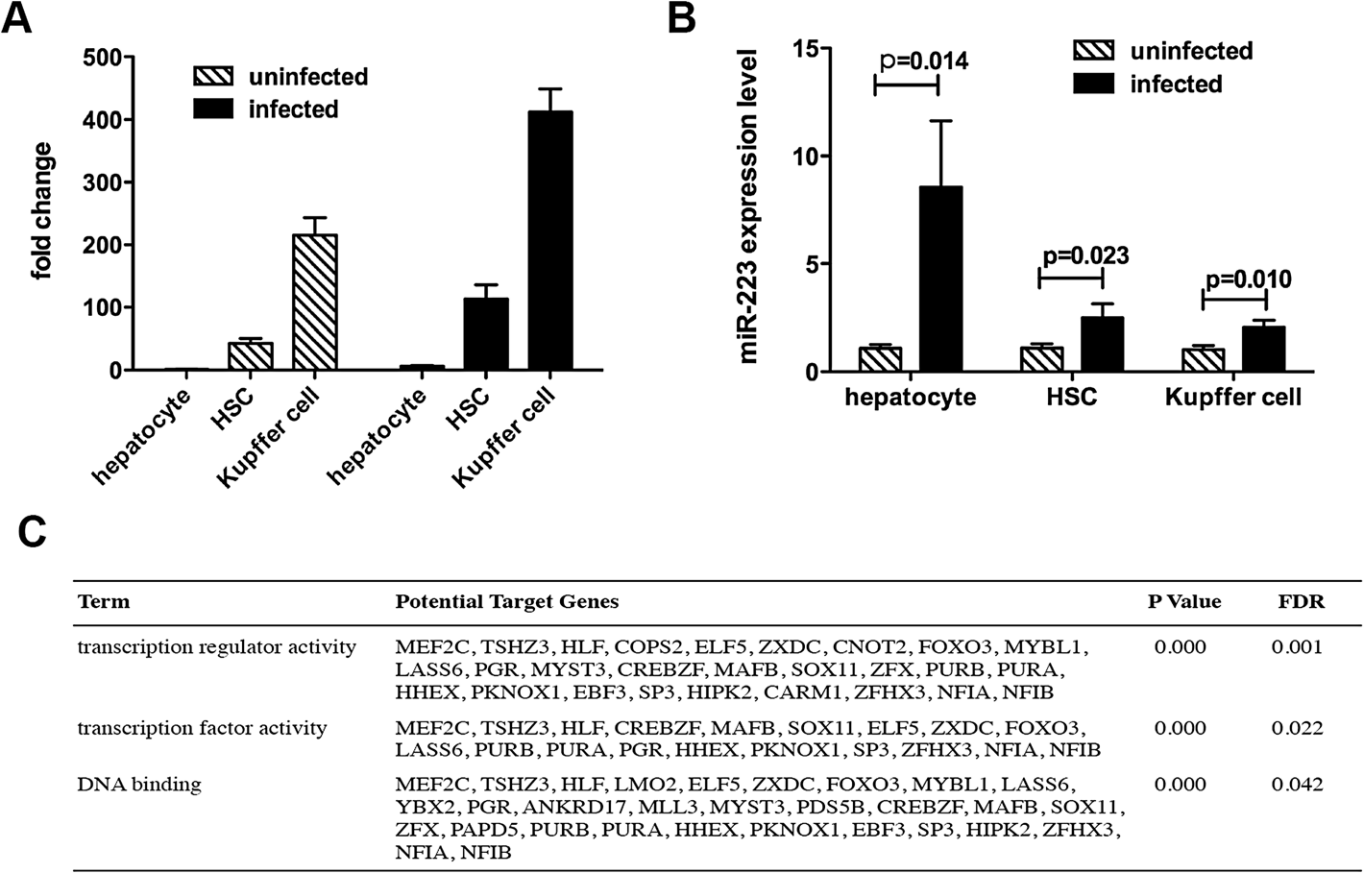
**Expression level of miR-223 in resident liver cells.** Mice were infected percutaneously with 16 *S. japonicum* cercariae or not infected as control, and sacrificed at 56 days after infection to isolate the hepatocytes, HSCs and Kupffer cells. Relative miR-223 expression in comparison with uninfected hepatocytes was determined by qPCR **(A)**, and the miR-223 expression levels of hepatocytes, HSCs and Kupffer cells after infection were analyzed **(B)**. The potential role of miR-223 was analyzed by bioinformatics analyses **(C)**.

We isolated the primary resident livers cells, including hepatocytes, hepatic stellate cells (HSCs) and Kupffer cells, to quantify the expression level of miR-223. We found that miR-223 was primarily located in the Kuppfer cells of both infected and uninfected livers (Figure 4A). However, a significant elevated expression level of miR-223 was detected in not only the Kuppfer cells, but also hepatocytes and HSCs after infection (Figure 4B). Bioinformatics analyses (TargetScan analysis [28] and Gene ontology analysis [29]) revealed a potential role of miR-223 in transcription regulator activity, transcription factor activity and DNA binding (Figure 4C).

## Discussion

Circulating miRNAs are stable even if subjected to harsh conditions, and their expression profiles are often altered in numerous human diseases including liver diseases [17]. These make them ideal candidates for use as biomarkers for disease onset and progression. Thus identification of the characteristically changed serum miRNAs in the pathogenesis and progression of human diseases has been used to identify new blood-based biomarkers for disease diagnosis and prognosis. Schistosomiasis is a worldwide parasitic disease that causes liver fibrosis and ultimately the death of the host. Currently, there are still no reliable biomarkers for the early diagnosis of schistosomiasis or the detection of disease progress after administration of therapeutic drugs, such as PZQ.

In this study, we evaluated the levels of six candidate serum miRNAs (known to be commonly deregulated in human liver diseases) in multiple host species infected with *S. japonicum*. Using a mouse model of schistosomiasis japonica, we found that the expression level of serum miR-223 was significantly elevated after infection, yet returned to near normal levels after treatment with the anti-helminth drug PZQ. Importantly, we found that the level of serum miR-223 reflected the extent of liver pathology post-infection. In addition, we observed that circulating miR-223 was also significantly elevated in other hosts infected with *S. japonicum*, including rabbits, buffalos and humans, validating the results of the murine model. It is true that the elevated level of the serum miR-223 was associated with schistosomiasis, but it may not be specifically associated with the disease because the elevated level of the miR-223 was also observed in chronic hepatitis [18]. The explanation could be that alteration of a circulating miRNA can be indicative of pathological changes for some diseases sharing some common pathogenesis processes such as inflammation, immune response, or fibrosis etc. Thus, those individual circulating miRNAs can serve as the biomarkers for those diseases sharing some common pathogenesis processes. For examples, serum miR-155 is the potential biomarker for B-cell lymphoma [30] but also for other cancers such as breast cancer [31], ovarian cancer [32], and pancreatic cancer [33]; Serum miR-92 is the potential biomarker for colon cancer [34], but also leukemia [35], and ovarian cancer [32]; Circulating miR-223 is the potential biomarker for chronic hepatitis [18], but also for schistosomiasis observed in this study. Due to currently lack of the effective tools for detection of schistosome infection, the serum miR-223 identified in this study should be useful in combination with other tools such as immunodiagnostic assays or other molecular markers to be identified and available for epidemiological information for the detection of schistosome infection.

In addition to the association of the serum miR-223 with schistosomiasis, we also showed that the levels of the circulating miR-223 were significantly declined and returned to normality after treatment of the host with the anti-helminth drug, praziquantel. This finding is most interesting because it is specifically associated with the extent of hepatic schistosomiasis and has an important potential prospect for application. The schistosome parasites live in the mesenteric veins. Currently there is no specific molecular tool available to detect the efficacy of the anti-helminth drug, PZQ. Development of such a tool is extremely important for effectively targeting treatment to all the infected hosts and currently the schistosomiasis elimination program is based on the control of infection sources. We showed both hydroxyproline content and the circulating miR-223 were returned to near normality within one month after PZQ treatment, indicating the serum miR-223 marker is sensitive to chemotherapy and could be potentially developed as new tool for measurement of the response to chemotherapy.

It is widely accepted that circulating miRNAs are derived either via passive release from injured tissues [36] or via active release from cells through microvesicles [37]. In this study, we isolated the primary hepatocytes, HSCs and Kupffer cells from the infected and uninfected livers to quantify the expression level of miR-223. We found miR-223 was primarily located in the Kupffer cells, and the expression level of miR-223 was significantly up-regulated in all these three types of resident liver cells post-infection, which suggests that the elevated serum miR-223 is derived from the infected liver. Nowadays, it is well acknowledged that the regulation and function of miRNAs is cell-type specific. Considering the Kupffer cells are essential for the development of schistosomiasis-induced inflammation and fibrosis [38], our data implied that miR-223 could play an important role in the pathogenesis and progression of schistosomiasis japonica via regulating the function of Kupffer cells. Importantly, bioinformatics analyses showed that miR-223 potentially functions in transcription regulator activity, transcription factor activity and DNA binding. This suggested that miR-223 could regulate the transcription of some important genes in the Kupffer cells to modulate their function. Taken together, our data proved that miR-233 is a schistosomiasis-associated miRNA.

## Conclusions

These results indicate that miR-233 was a schistosomiasis-associated miRNA, and thus circulating miR-223 may serve as a new potential biomarker for the detection of schistosome infection and the assessment of the response to chemotherapy.

## Competing interests

The authors declare that they have no competing interests.

## Authors’ contributions

XH, XS, DMZ and WQP conceived and designed the study. XH, XS, CC, YBZ, and XDX performed the experiments and analyzed the data. XH and WQP wrote the manuscript. All authors read and approved the final manuscript.
